# Long noncoding RNA TFAP2A-AS1 exerts promotive effects in non-small cell lung cancer progression via controlling the microRNA-548a-3p/CDK4 axis as a competitive endogenous RNA

**DOI:** 10.32604/or.2022.03563

**Published:** 2022-07-13

**Authors:** YANG ZHANG, LIXIA MA, TINGTING ZHANG, PEIDONG LI, JIABIN XU, ZHUO WANG

**Affiliations:** Department of Oncology, Jilin Province Cancer Hospital, Jilin, 130012, China

**Keywords:** TFAP2A antisense RNA 1, Lung cancer, ceRNA pathway, Therapeutic target

## Abstract

In this study, we mainly focus on probing expression profile and detailed functions of long non-coding RNA TFAP2A antisense RNA 1 (TFAP2A-AS1) in non-small cell lung cancer (NSCLC). Moreover, the mechanisms played by TFAP2A-AS1 were unraveled comprehensively. Herein, a notable overexpressed TFAP2A-AS1 in NSCLC was observed by TCGA and our own cohort. An increased TFAP2A-AS1 level displayed a negative correlation with the overall survival of patients with NSCLC. Loss-of-function approaches illustrated that the absence of TFAP2A-AS1 weakened NSCLC cell proliferation, colony formation, migration and invasion *in vitro*. Also, interference of TFAP2A-AS1 caused *in vivo* tumor growth suppression. Mechanistically, TFAP2A-AS1 could negative regulate microRNA-584-3p (miR-584-3p) as a competitive endogenous RNA. Furthermore, cyclin-dependent kinase 4 (CDK4), a direct target of miR-584-3p, was positively controlled by TFAP2A-AS1 in a miR-5184-3p-dependent manner. Rescue function experiments corroborated that the anticancer activities of TFAP2A-AS1 deficient on the oncogenicity of NSCLC cells were reversed by downregulating miR-584-3p or overexpressing CDK4. To sum up, TFAP2A-AS1 exhibits cancer-promoting roles in NSCLC through the adjustment of miR-584-3p/CDK4 axis.

## Introduction

Lung cancer ranks as the most prevailing human cancer and is the principal reason of tumor-involved deaths around the world [1]. As a predominant type of lung cancer, non-small cell lung cancer (NSCLC) occupies about 80%–85% of all human lung cancer cases, and over 70% of NSCLC cases have developed into middle or advanced stage when symptoms emerge [2]. Since significant progress in terms of basic research, the primary treatment approach, including minimally invasive surgery, radiochemotherapy and targeted therapy, have made commendable advancement in the past decade [3,4]; yet, the clinical efficiency of NSCLC patients remains gloomy [5]. The 5-year survival rate of NSCLC patients at early stage is about 50%, but decreases to a level lower than 5% [6]. Therefore, making great efforts on the mechanisms of NSCLC pathogenesis may help for the identification of promising diagnostic and therapeutic targets.

Long non-coding RNAs (lncRNAs) are described as untranslated transcripts with more than 200 nucleotides [7]. Although they cannot be translated to protein, lncRNAs play a part in the control of genes expression that exert essential roles in mounting physiological and pathological cellular processes [8]. Striking, the expression status of lncRNA presents a notable correlation with a range of human diseases [9]. A substantial number of lncRNAs are deregulated in NSCLC, and contribute to diversified steps during cancer genesis and progression [10–12]. The competitive endogenous RNA (ceRNA) theory depicts that lncRNAs have the power to sequester microRNAs (miRNAs), and kept miRNAs away from their target mRNAs, consequently constituting a regulatory network [13]. Thus, lncRNAs and miRNAs have a perfect prospect in the development of novel therapeutic strategies for NSCLC.

Utilizing the cancer genome atlas (TCGA) dataset, we found that TFAP2A antisense RNA 1 (TFAP2A-AS1) was highly expressed in almost all human cancer types, including lung squamous cell carcinoma (LUSC) and lung adenocarcinoma (LUAD). Thus, we mainly focus on probing expression profile and detailed functions of TFAP2A-AS1 in NSCLC. Moreover, the molecular events occurred by TFAP2A-AS1 were unraveled comprehensively.

## Materials and Methods

### Patients and cell lines

The current research conformed to the Ethical Committee of Jilin Province Cancer Hospital, and was carried out in accordance with the Declaration of Helsinki. After getting the written inform consents, NSCLC tissues were collected from 47 patients with NSCLC in Jilin Province Cancer Hospital. Meanwhile, corresponding adjacent normal tissues were obtained and served as the control.

The inclusion criteria were as follows: (i) patiens who were diagnosed as NSCLC; (ii) was not treated with systemic or local anticancer treatments before the application of surgery; and (iii) agreed to take part in the study. The exclusion criteria were as follows: (i) patients with systemic or local anticancer treatments before surgery; and (ii) did not agree to take part in the study. All samples were stored in liquid nitrogen till needed.

BEAS-2B is a human nontumorigenic bronchial epithelial cell line and cultured in Bronchial epithelial cell growth medium (Lonza, Walkersville, MD, USA) supplemented with 10% fetal bovine serum (FBS) and 1% penicillin-streptomycin solution (Gibco; Thermo Fisher Scientific, Inc., Waltham, MA, USA). NSCLC cell lines, A549 and H1299, were grown in RPMI-1640 medium (Gibco). H460 and SK-MES-1, another two NSCLC cell lines, were maintained in F-12K and MEM (Gibco), respectively. Beyond that, the culture conditions of these NSCLC cell lines were the same as those for BEAS-2B. All cell lines used were bought from American Type Culture Collection (Manassas, VA, USA), and grown at 37°C in a humidified incubator containing 5% CO_2_.

### Transfection

GenePharma Co., Ltd. (Shanghai, China) designed and synthesized the TFAP2A-AS1-targeted small interfering RNA (siRNA; si-TFAP2A-AS1) and negative control (NC) siRNA (si-NC). Cyclin-dependent kinase 4 (CDK4) overexpression plasmid pcDNA3.1-CDK4 was also generated by GenePharma Co.,Ltd. MiRNA oligonucleotides, including miR-584-3p mimic, NC mimic, miR-584-3p inhibitor (anti-miR-584-3p) and NC inhibitor (anti-NC), were offered by RiboBio (Guangzhou, China). Cells were seeded into 6-well plates and grown to 70%–80% confluency. Cellular transfection was accomplished employing Lipofectamine 2000 (Invitrogen). Quantitative real-time polymerase chain reaction (qRT-PCR) was employed to appraise transfection efficiency.

### qRT-PCR

After being extracted utilizing Trizol (Beyotime; Shanghai, China), total RNA was quantified with a NanoDrop 1000 spectrophotometer (NanoDrop Technologies; Thermo Fisher Scientific, Inc.). For the determination of TFAP2A-AS1 and CDK4 expression, reverse transcription was operated utilizing the PrimeScript^™^ RT reagent kit with gDNA Eraser (Takara; Dalian, China) to produce complementary DNA. PCR amplification was then implemented with a TB Green® Premix Ex Taq^™^ II (Takara). Glyceraldehyde-3-phosphate dehydrogenase (GAPDH) functioned as the reference control for TFAP2A-AS1 and CDK4. For miR-584-3p quantification, reverse transcription and PCR amplification were undertaken applying miScript Reverse Transcription kit and miScript SYBR Green PCR kit (Qiagen GmbH, Hilden, Germany), respectively. MiR-584-3p was normalized to small nuclear RNA U6. The 2^−ΔΔCq^ formula was used for genes expression calculation.

### Cell counting kit-8 (CCK-8) assay

Transfected cells were harvested at 24 h post-transfection, and mixed with complete culture medium to obtain cell suspension. After adjusting the concentration of cell suspension to 2 × 10^4^ cells/mL, every well of 96-well plates was covered with 100 µl cell suspension. Prior to being treated with 10 µl CCK-8 solution (Beyotime), cells were cultivated at 37°C with CO_2_ for different time periods. Following additional 2 h incubation, the optical density at 450 nm wavelength (OD450) was monitored by means of a microplate reader.

### Colony formation assay

The harvested cells were resuspended in complete culture medium, and then inoculated into 6-well plates. Each well was covered with 2 ml cell suspension containing 500 cells. After culturing for 14 days, the culture medium was discarded, and formed colonies were fixed with 4% paraformaldehyde, and stained with 0.1% crystal violet. Finally, the colonies were photographed applying an inverted microscope (Olympus Corp., Tokyo, Japan).

### Transwell assay

The Matrigel precoated transwell insets (BD Biosciences) were applied for invasion test. A total of 4  × 10^4^ cells resuspended in 200 µl FBS-free medium were plated into the upper compartments. By acting as a chemoattractant, the lower compartments were fulfilled with 600 µl culture medium which was added with 20% FBS. After culturing at 37°C for 24 h, the upper surface of membranes was scrubbed with a cotton swab, whereas the invaded cells were fixed with 4% paraformaldehyde and dyed with 0.1% crystal violet. After snapping with a light microscope (×200 magnification), the amount of invaded cells was counted in five randomly chosen visuals. The uncoated transwell insets were applied in migration test, and followed the same instructions.

### Tumor xenograft experiment

The animal procedures conformed to the Animal Care Committee of Jilin Province Cancer Hospital, and were implemented in line with NIH guidelines for the care and use of laboratory animals. The TFAP2A-AS1-targeted short hairpin RNA (shRNA; sh-TFAP2A-AS1) and NC shRNA (sh-NC; GenePharma Co., Ltd., China) were inserted into the lentivirus vectors, after which were transfected into 293T cells (Cell Bank of the Chinese Academy of Sciences; Shanghai, China). 293T cells were cultured in DMEM medium that was added with 10% FBS, 1% Glutamax and 1% Sodium Pyruvate (all from Gibco). The virus supernatant was harvested after 48 h cultivation, and was utilized to infect A549 cells. The stable TFAP2A-AS1-ablated cell line was screened out by treating with puromycin.

Male BALB/c nude mice (Shanghai Experimental Animal Center; Shanghai, China), aged 4–6 weeks, were subcutaneously injected utilizing A549 cells overexpressing sh-TFAP2A-AS1 or sh-NC. Tumor volume was recorded weekly by monitoring the width and length of subcutaneous xenografts, and calculated with the formula: volume = 1/2 × length × width^2^. The observation lasted for 4 weeks. As soon as the experiment ends, mice were euthanized, and the tumor xenografts were excised for weighing.

### Subcellular fractionation

A Cytoplasmic & Nuclear RNA Purification Kit (Norgen, Belmont, CA) was applied for cell cytoplasm and nucleus separation. qRT-PCR was conducted to determine the TFAP2A-AS1 abundance in cytoplasmic and nuclear RNA.

### Bioinformatics analysis

miRDB (http://mirdb.org/) was employed to find the downstream target of TFAP2A-AS1. The targeting relationship between miR-584-3p and CDK4 3’-UTR was predicted with the application of TargetScan (http://www.targetscan.org/) and miRDB.

### Luciferase reporter assay

TFAP2A-AS1 and CDK4 3’-UTR sequences containing wild-type (wt) miR-584-3p binding site were amplified and cloned into the pmirGlO dual-luciferase Vector (Promega, Madison, WI, USA). The constructed luciferase reporter vectors were named as wt-TFAP2A-AS1 and wt-CDK4. In the meantime, the same steps were followed and established the mutated (mut)-TFAP2A-AS1 (mut-TFAP2A-AS1) and mut-CDK4 reporter vectors comprising mutant miR-584-3p binding site. The transfection of wt or mut reporter vectors in parallel with miR-584-3p mimic or NC mimic was accomplished applying Lipofectamine 2000. As per user manual, luciferase activity was measured utilizing a Dual-Luciferase Reporter Assay System (Promega) at 48 h post-transfection.

### RNA immunoprecipitation (RIP)

RIP assay was executed to delve into the interaction among TFAP2A-AS1, miR-584-3p and CDK4. The whole cell extract was prepared by lysing cells with RIP lysis buffer came from a Magna RIP RNA Binding Protein Immunoprecipitation Kit (Millipore, Darmstadt, Germany). RIP buffer supplemented with magnetic beads coupled to Argonaut 2 (Ago2) or normal mouse IgG (Millipore) antibodies was adopted to treat the cell lysate at 4°C the whole night. The magnetic beads were collected, probed with Protease K, and were processed for immunoprecipitated RNA extraction. Relative enrichment was examined via qRT-PCR.

### Western blotting

After being abstracted utilizing RIPA lysis buffer, the concentration of total protein was detected with a BCA kit (both from Beyotime). Equivalent proteins were divided by 10% SDS-PAGE electrophoresis, and then transferred onto PVDF membranes. Tris Buffered Saline with Tween® 20 supplemented with 5% nonfat dried milk was applied for blocking the membranes at room temperature for 2 h. In the next step, the membranes were hatched all night at 4°C with the primary antibodies targeting CDK4 (ab108357; dilution 1:1000) or GAPDH (ab128915; dilution 1:1000; Abcam, Cambridge, MA, USA). Subsequent to three washes utilizing Tris Buffered Saline with Tween® 20, the membranes were received cultivation with horseradish peroxidase-conjugated secondary antibody (ab205718; dilution 1:4000; Abcam) and further visualization using Pierce^™^ ECL Western Blotting Substrate (Thermo Fisher Scientific). Quantity One software (Bio Rad Laboratories, Inc., Hercules, CA, USA) was adopted for analyzing the protein signals.

### Statistical analysis

All experiments were repeated for three times. The measurement data are represented as mean ± standard deviation. Survive curves were plotted with the application of Kaplan-Meier method, and compared applying log-rank test. Correlation analysis was conducted utilizing Pearson correlation coefficient. One-way analysis of variance was used in the comparison among multiple groups. Tukey’s test was carried for *post hoc* test. The comparison between two groups was fulfilled via Student’s *t*-test. All statistical analysis was accomplished with the help of SPSS 19.0 (SPSS, Chicago, IL, USA). There was a statistical difference when *p* < 0.05.

## Results

### Overexpressed TFAP2A-AS1 is detected in NSCLC

**Figure 1:**
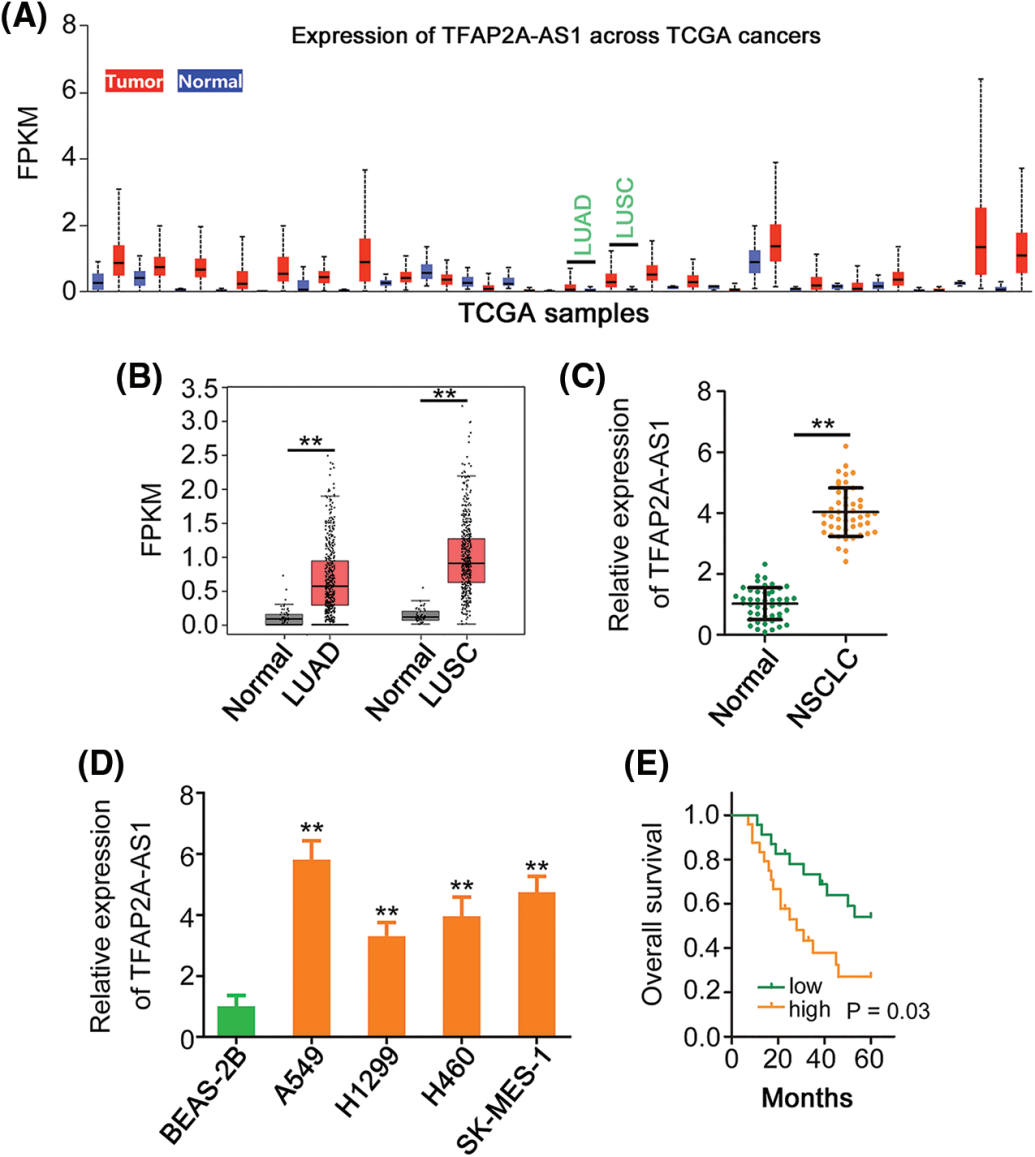
TFAP2A-AS1 is upregulated in NSCLC. (A) Expression profile of TFAP2A-AS1 across TCGA cancers. (B) TFAP2A-AS1 level in LUAD and LUSC was examined in TCGA. (C) Detection of TFAP2A-AS1 expression in NSCLC tissues was conducted via qRT-PCR. (D) Determination of TFAP2A-AS1 expression in NSCLC cell lines was implemented via qRT-PCR. (E) Kaplan–Meier analysis illustrated the correlation between TFAP2A-AS1 expression and overall survival in patients with NSCLC. **p < 0.01.

Through TCGA dataset, we found that TFAP2A-AS1 was highly expressed in almost all human cancer types (Fig. 1A), also including LUSC and LUAD (Fig. 1B). Next, expression of TFAP2A-AS1 was tested in NSCLC tissues, and TFAP2A-AS1 was upregulated in NSCLC tissues from our own cohort (Fig. 1C). Furthermore, highly expressed TFAP2A-AS1 was proved in the four tested NSCLC cell lines (Fig. 1D). We then executed survival analysis, and classified all 47 patients into either TFAP2A-AS1-low (*n* = 23) or TFAP2A-AS1-high (*n* = 24) groups. When compared with TFAP2A-AS1-low group, patients characterized by a high TFAP2A-AS1 level featured shorter overall survival (Fig. 1E).

### Knocking down TFAP2A-AS1 restrains the aggressive phenotype of NSCLC cells in vitro

**Figure 2:**
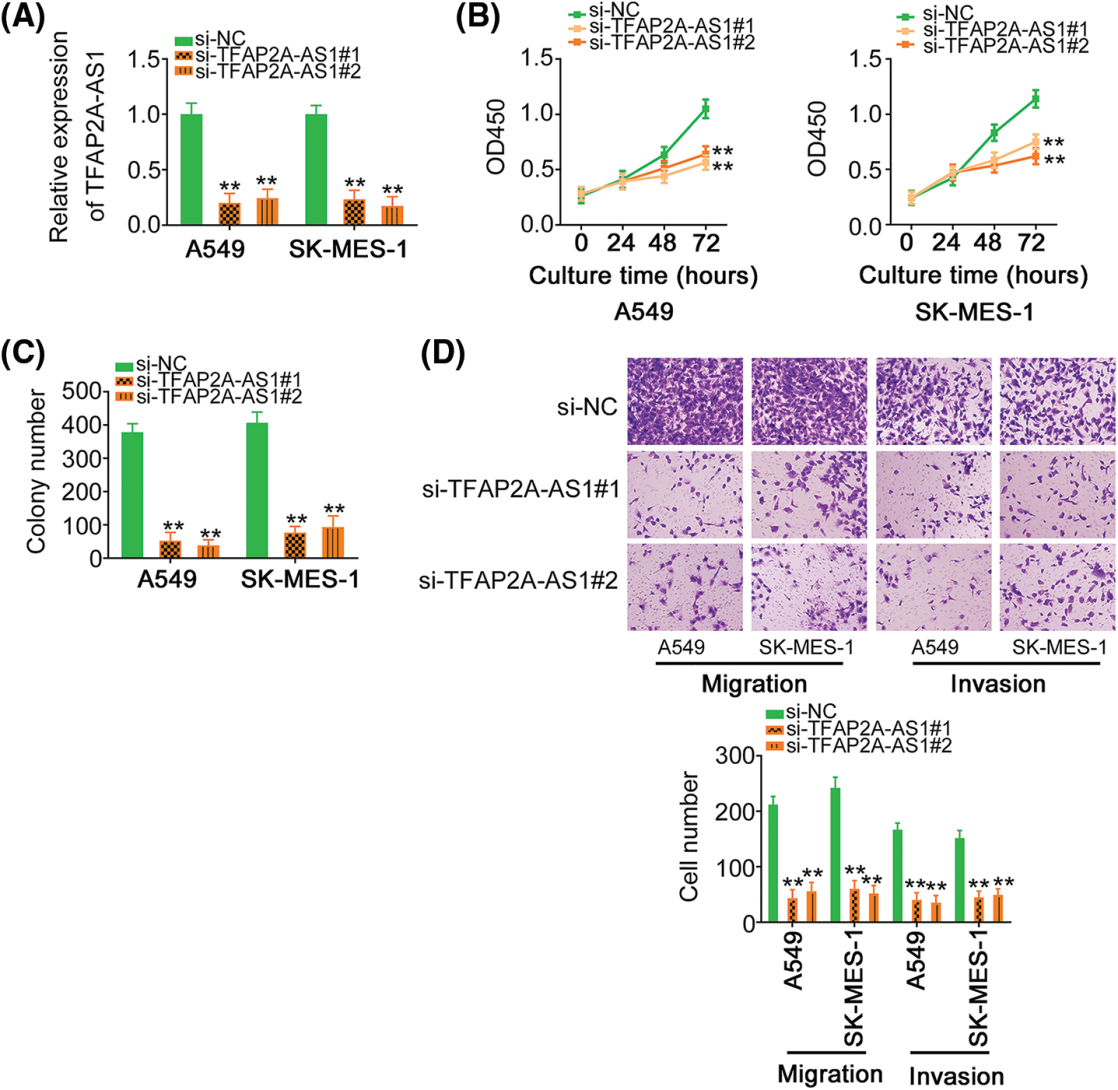
Depletion of TFAP2A-AS1 exerts repressing effects in NSCLC cells. (A) qRT-PCR was utilized for detecting TFAP2A-AS1 level in NSCLC cells following si-TFAP2A-AS1 transfection. (B) The proliferation of TFAP2A-AS1-silenced NSCLC cells was tested via CCK-8 assay. (C) The colony formation of NSCLC cells was detected when TFAP2A-AS1 was ablated. (D) Transwell assay was adopted to evaluate the impact of si-TFAP2A-AS1 on NSCLC cell motility (×200 magnification). **p < 0.01 (n = 3).

To ascertain whether TFAP2A-AS1 functionally contributed to NSCLC progression, knocking down TFAP2A-AS1 was occurred in A549 and SK-MES-1 cells by transfecting with si-TFAP2A-AS1 (Fig. 2A). The proliferation (Fig. 2B) and colony formation (Fig. 2C) of NSCLC cells was suppressed by TFAP2A-AS1 deficient. Furthermore, the migration and invasion of NSCLC cells was apparently suppressed in response to TFAP2A-AS1 ablation (Fig. 2D). Altogether, TFAP2A-AS1 plays a carcinogenic role in NSCLC cells.

### TFAP2A-AS1 exerts as a ceRNA for miR-584-3p and thereby positively controls CDK4 level

**Figure 3:**
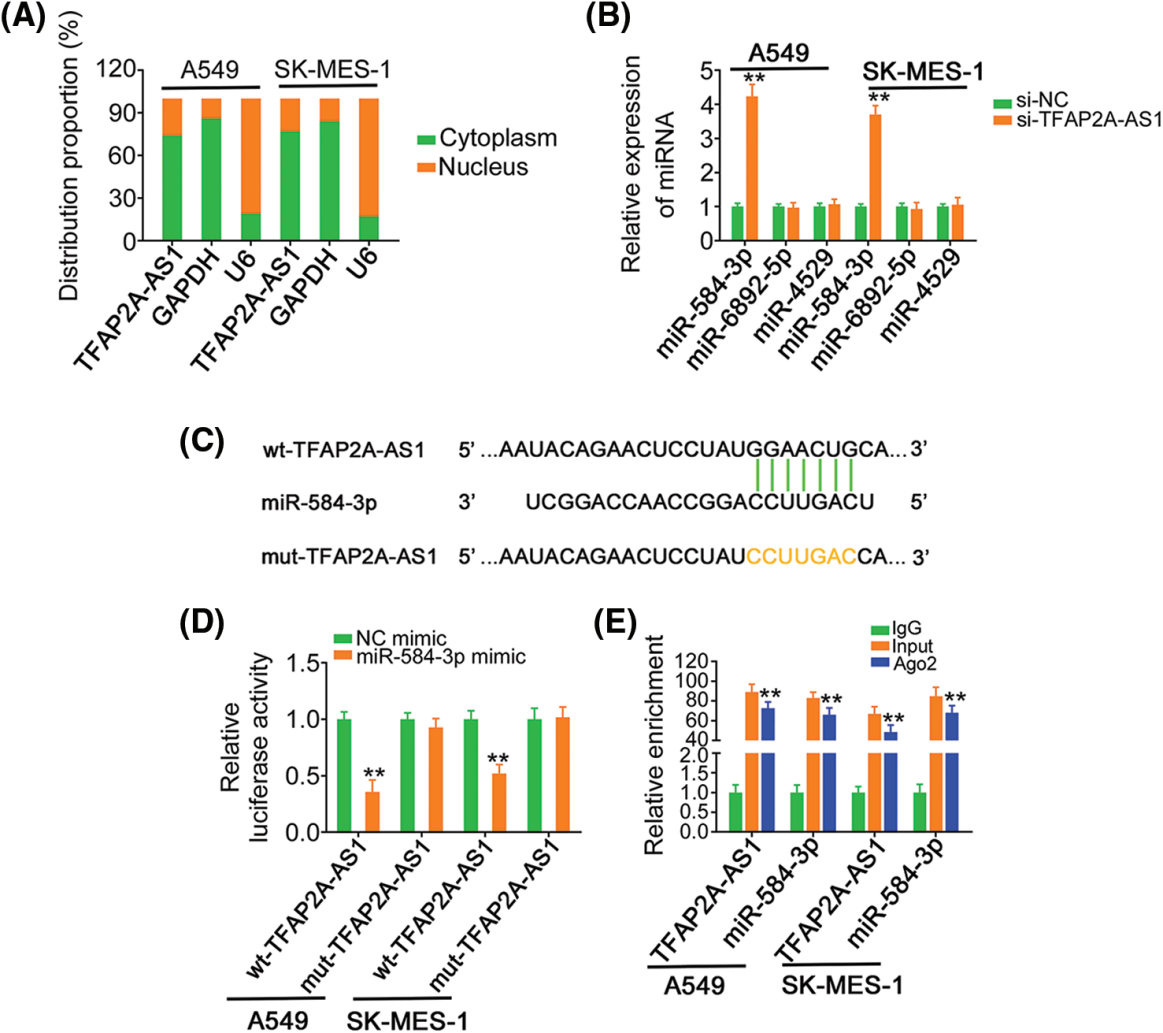
MiR-584-3p is sponged by TFAP2A-AS1 in NSCLC. (A) The distribution of TFAP2A-AS1 in NSCLC cells was illustrated by subcellular fractionation. (B) TFAP2A-AS1 was depleted in NSCLC cells, and then subjected to qRT-PCR for the measurement of miR-6892-5p, miR-4529, and miR-584-3p levels. (C) The predicted binding sequences between miR-584-3p and TFAP2A-AS1. The mutant site was also shown. (D) Luciferase activity of wt-TFAP2A-AS1 or mut-TFAP2A-AS1 was detected in NSCLC cells after being cotransfected with miR-584-3p mimic or NC mimic. (E) The direct interaction between miR-584-3p and TFAP2A-AS1 was certified applying RIP assay. **p < 0.01 (n = 3).

To define the working mechanisms used by TFAP2A-AS1, we firstly employed lncLocator (http://www.csbio.sjtu.edu.cn/bioinf/lncLocator/) to predict the subcellular location of TFAP2A-AS1. TFAP2A-AS1 was predicted to be chiefly distributed in cell cytoplasm (Table 1), which was further verified by subcellular fractionation experiment (Fig. 3A). The observation implied that TFAP2A-AS1 may perform its action in a miRNA-mRNA dependent manner. Utilizing miRDB, 30 miRNAs owned conserved binding sites for TFAP2A-AS1 (Table 2). Employing TCGA database, miR-6892-5p, miR-4529, and miR-584-3p are revealed to be downregulated in NSCLC, and therefore were deeply verified by a series of mechanistic experiments.

**Table 1:** The result of lncLocator prediction

Subcellular locations	Score
Cytoplasm	0.554348074121
Nucleus	0.134693060525
Ribosome	0.0427315963693
Cytosol	0.177283923386
Exosome	0.0909433455989

**Table 2:** The potential targets of TFAP2A-AS1 predicted by miRDB

Target rank	miRNA name
1	has-miR-4783-3p
2	has-miR-876-3p
3	has-miR-7846-3p
4	has-miR-4425
5	has-miR-6510-5p
6	has-miR-576-5p
7	has-miR-6892-5p
8	has-miR-4768-3p
9	has-miR-5088-3p
10	has-miR-4529-3p
11	has-miR-6774-3p
12	has-miR-6758-5p
13	has-miR-4256
14	has-miR-11181-5p
15	has-miR-5572
16	has-miR-584-3p
17	has-miR-518c-3p
18	has-miR-6765-5p
19	has-miR-6856-5p
20	has-miR-377-3p
21	has-miR-6780b-3p
22	has-miR-203b-3p
23	has-miR-4516
24	has-miR-4742-3p
25	has-miR-3191-5p
26	has-miR-580-3p
27	has-miR-6762-5p
28	has-miR-4433a-5p
29	has-miR-6845-3p
30	has-miR-4293

We then depleted TFAP2A-AS1 expression in NSCLC cells, and determined the expression change of the three miRNAs. Data suggested that only miR-584-3p level was increased in si-TFAP2A-AS1-transfected NSCLC cells (Fig. 3B). Next, the direct binding between TFAP2A-AS1 and miR-584-3p (Fig. 3C) was certified via luciferase reporter assay. As depicted in Fig. 3D, miR-584-3p downregulated the luciferase activity of wt-TFAP2A-AS1 in NSCLC cells, whereas the repressing activity was lost in response to mut-TFAP2A-AS1. Further, when compared with IgG control, TFAP2A-AS1 and miR-584-3p were striking enriched in Ago2 group (Fig. 3E). Therefore, TFAP2A-AS1 performed as a nature miRNA sponge for miR-584-3p in NSCLC.

**Figure 4:**
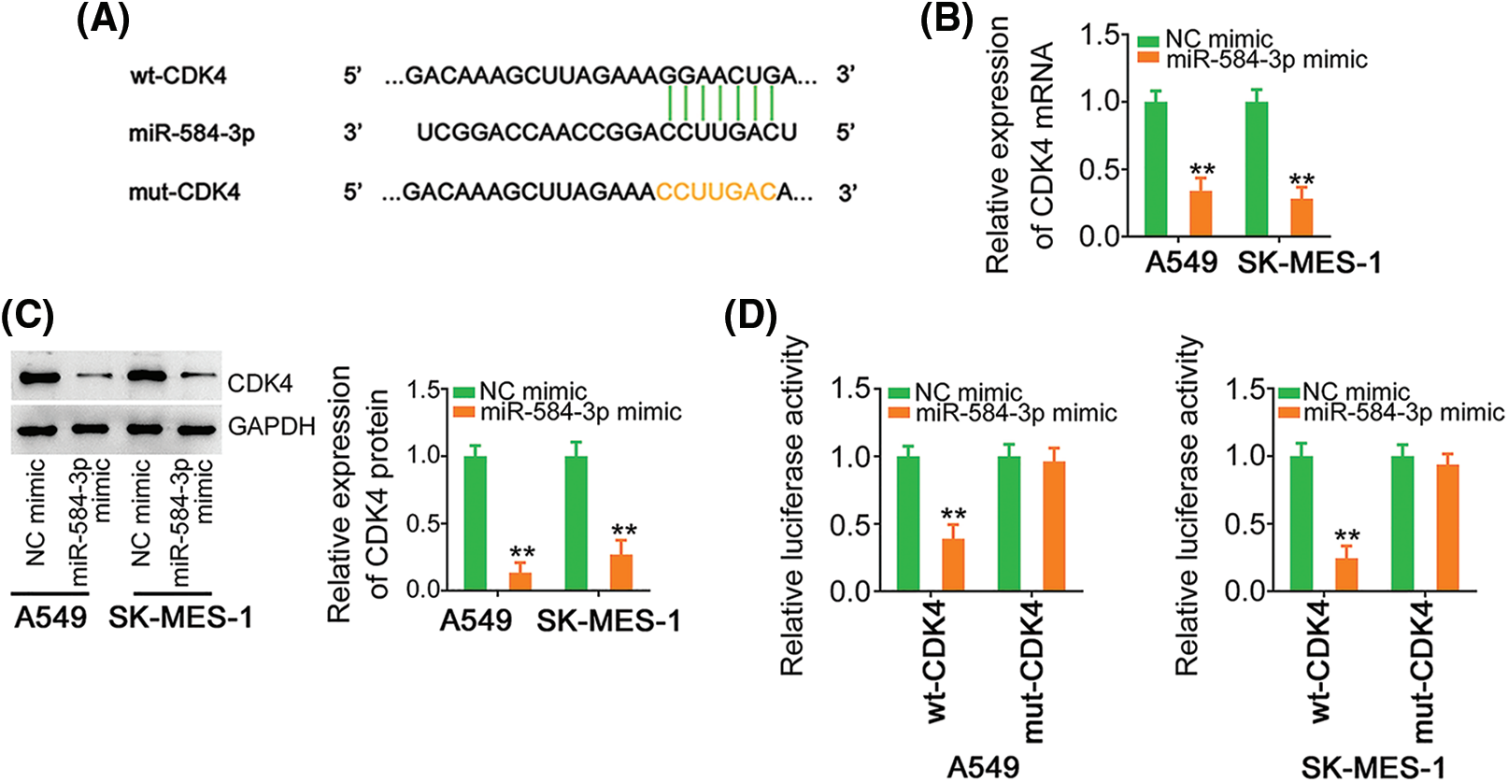
MiR-584-3p directly targets CDK4. (A) Schematic of the predicted target site for miR-584-3p within CDK4 3’-UTR and mutated binding site. (B, C) Levels of CDK4 in miR-584-3p-overexpressed NSCLC cells were determined utilizing qRT-PCR and western blotting. (D) Luciferase activity of wt-CDK4 or mut-CDK4 was detected in NSCLC cells after being cotransfected with miR-584-3p mimic or NC mimic. **p < 0.01 (n = 3).

**Figure 5:**
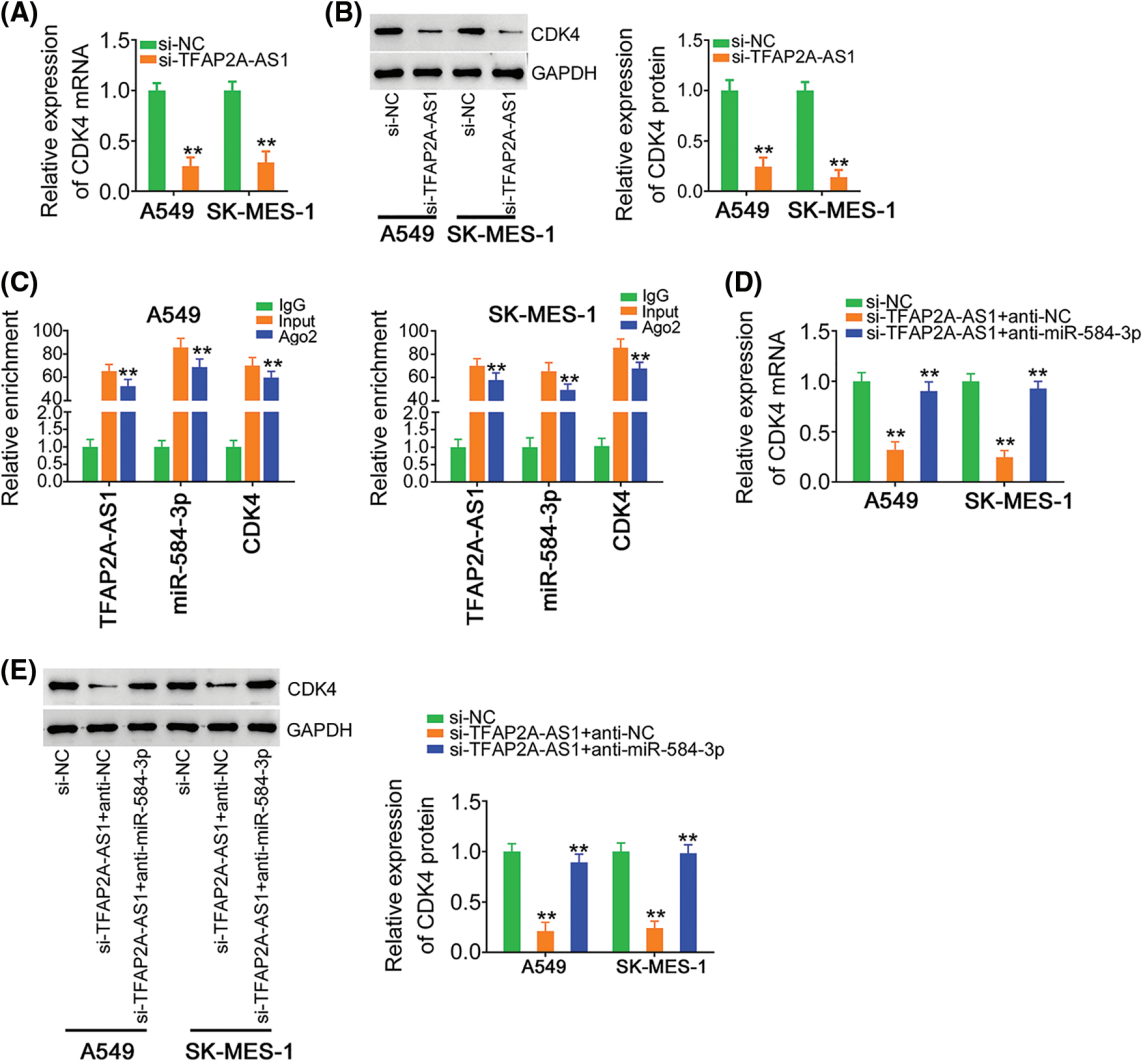
CDK4 is under control of TFAP2A-AS1/miR-584-3p in NSCLC cells. (A, B) Levels of CDK4 in TFAP2A-AS1-deficient NSCLC cells were determined utilizing qRT-PCR and western blotting. (C) The direct interaction between TFAP2A-AS1, miR-584-3p and CDK4 was certified applying RIP assay. (D, E) Anti-miR-584-3p was transfected into TFAP2A-AS1-silenced NSCLC cells, and then subjected to qRT-PCR and western blotting for CDK4 quantification. **p < 0.01 (n = 3).

MiRNAs work through affecting their target genes. Accordingly, bioinformatic analysis was conducted, and predicted CDK4 as a putative target of miR-584-3p (Fig. 4A). CDK4 performs carcinogenic actions during initation and progression during and is implicated in the regulation of tumor processes in NSCLC [14–16]. Therefore, CDK4 was selected for subsequent verification. We then confirmed that exogenous miR-584-3p caused a notable suppression of CDK4 expression (Figs. 4B and 4C). Furthermore, the treatment of miR-584-3p mimic lowered the activity of wt-CDK4; but, overexpressed miR-584-3p was unsuccessful in modulating the activity of reporter vector comprising mutant miR-584-3p binding site (Fig. 4D). After certifying CDK4 as a direct target of miR-584-3p, we next intended to clarify whether TFAP2A-AS1 affected CDK4 level in NSCLC. Expression of CDK4 was observed to be lowered by TFAP2A-AS1 deficient in NSCLC cells (Figs. 5A and 5B). Additionally, as relative to IgG control, TFAP2A-AS1, miR-584-3p and CDK4 were all were enriched after Ago2 treatment (Fig. 5C). Furthermore, the downregulated CDK4 by si-TFAP2A-AS1 was restored in NSCLC cells after anti-miR-584-3p cotransfection (Figs. 5D and 5E). In short, the described findings confirm that TFAP2A-AS1 functions as a ceRNA and decoys miR-584-3p, consequently positively modulating CDK4.

### MiR-584-3p upregulation decreases the malignancy of NSCLC cells

**Figure 6:**
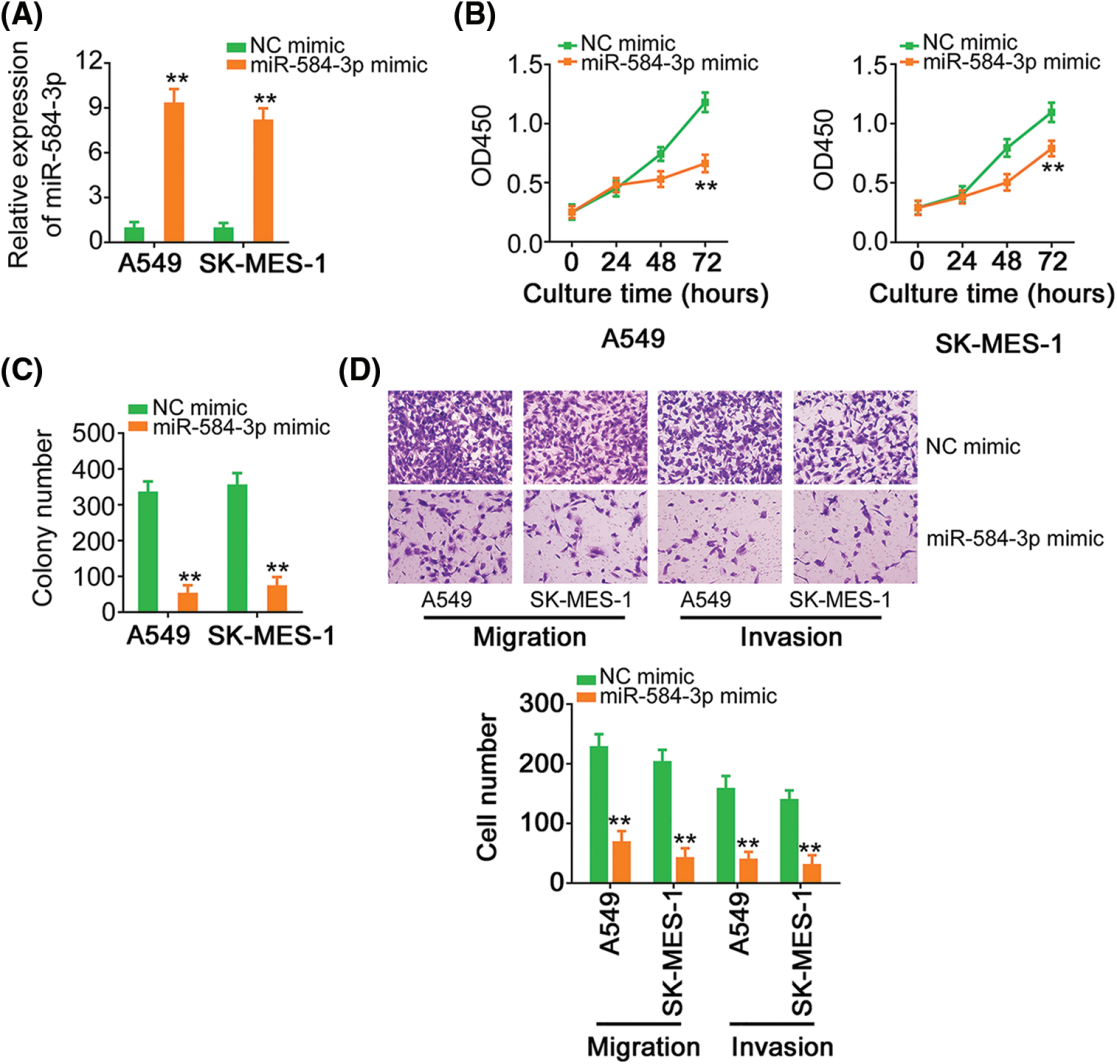
MiR-584-3p upregulation restricts the oncogenicity of NSCLC cells. (A) The efficiency of miR-584-3p mimic transfection was confirmed by qRT-PCR. (B, C) The proliferation and colony formation was tested in NSCLC cells which were transfected with miR-584-3p mimic or NC mimic. (D) The migratory and invasive properties of miR-584-3p mimic-transfected NSCLC cells were detected via Transwell assay (×200 magnification). **p < 0.01 (n = 3).

To reveal the function of miR-584-3p, we upregulated its expression in NSCLC by transfecting with miR-584-3p mimic (Fig. 6A). In comparison with NC mimic-transfected cells, exogenous miR-584-3p impaired the proliferation (Fig. 6B) and colony formation capacity (Fig. 6C) of NSCLC cells. Besides, ectopic miR-584-3p expression damaged the migratory and invasive capacities of NSCLC cells (Fig. 6D).

### TFAP2A-AS1 deficient disrupts the oncogenicity of NSCLC cells through targeting miR-584-3p/CDK4 axis

**Figure 7:**
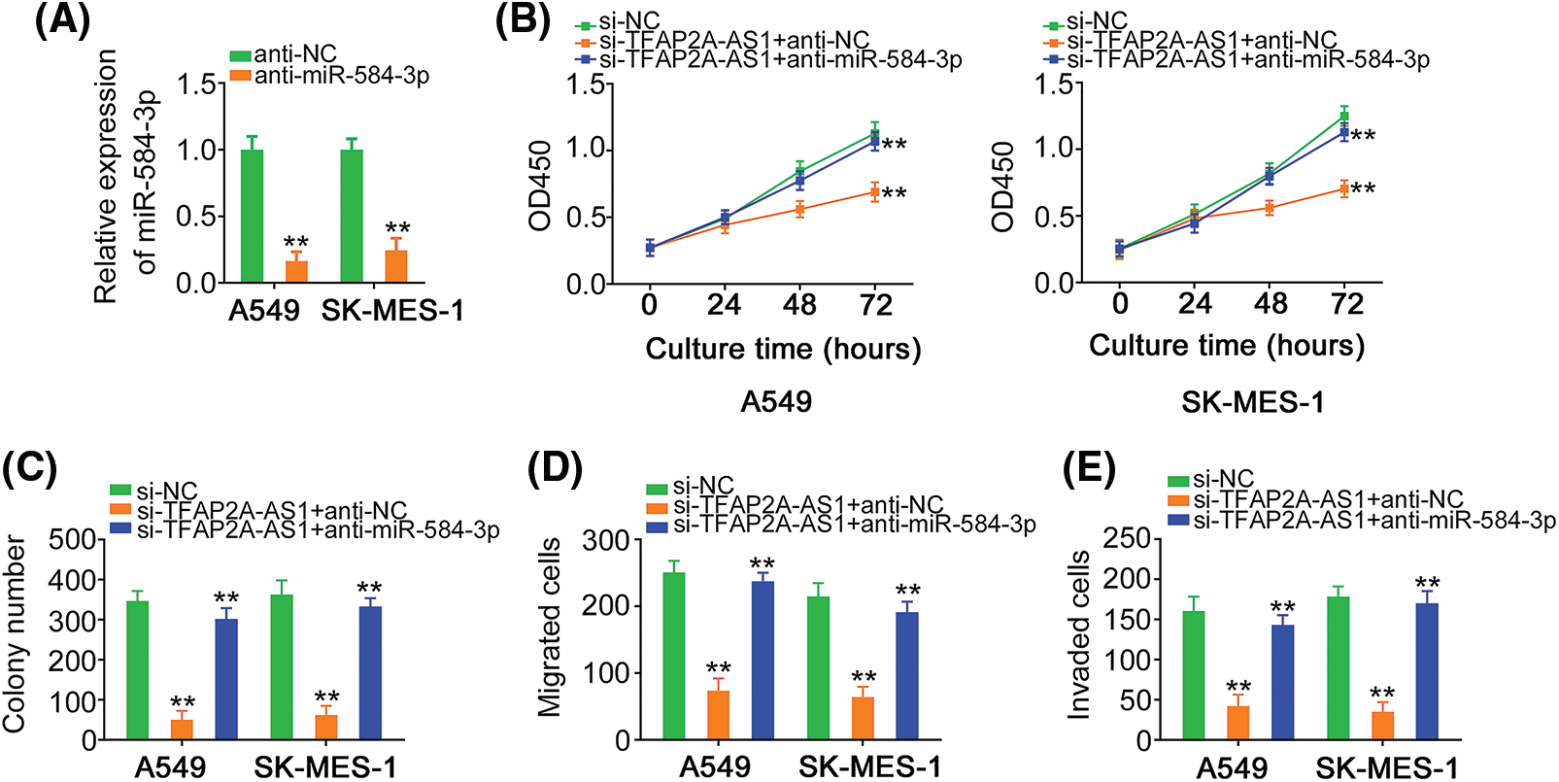
Downregulation of miR-584-3p confers the impacts of si-TFAP2A-AS1 on NSCLC cells. (A) The efficiency of anti-miR-584-3p transfection was proved by qRT-PCR. (B, C) anti-miR-584-3p or anti-NC alongside si-TFAP2A-AS1 was transfected into NSCLC cells. CCK-8 and colony formation assays were implemented to measure cell proliferation. (D, E) Transwell assay was conducted in the abovementioned cells for the determination of cell migration and invasion (×200 magnification). **p < 0.01 (n = 3).

In this final step, a string of recuse experiments were done to illustrate whether miR-584-3p/CDK4 axis is required for the biological activity of si-TFAP2A-AS1 in NSCLC. Prior to that, the interference efficiency of anti-miR-584-3p in NSCLC cells was verified via qRT-PCR (Fig. 7A). Ablation of TFAP2A-AS1 restricted cell proliferation and colony formation, in which treatment of anti-miR-584-3p counteracted the regulatory effects (Figs. 7B and 7C). In addition, si-TFAP2A-AS1 transfection triggered suppression on NSCLC cell motility was neutralized by inhibiting miR-584-3p (Figs. 7D and 7E).

**Figure 8:**
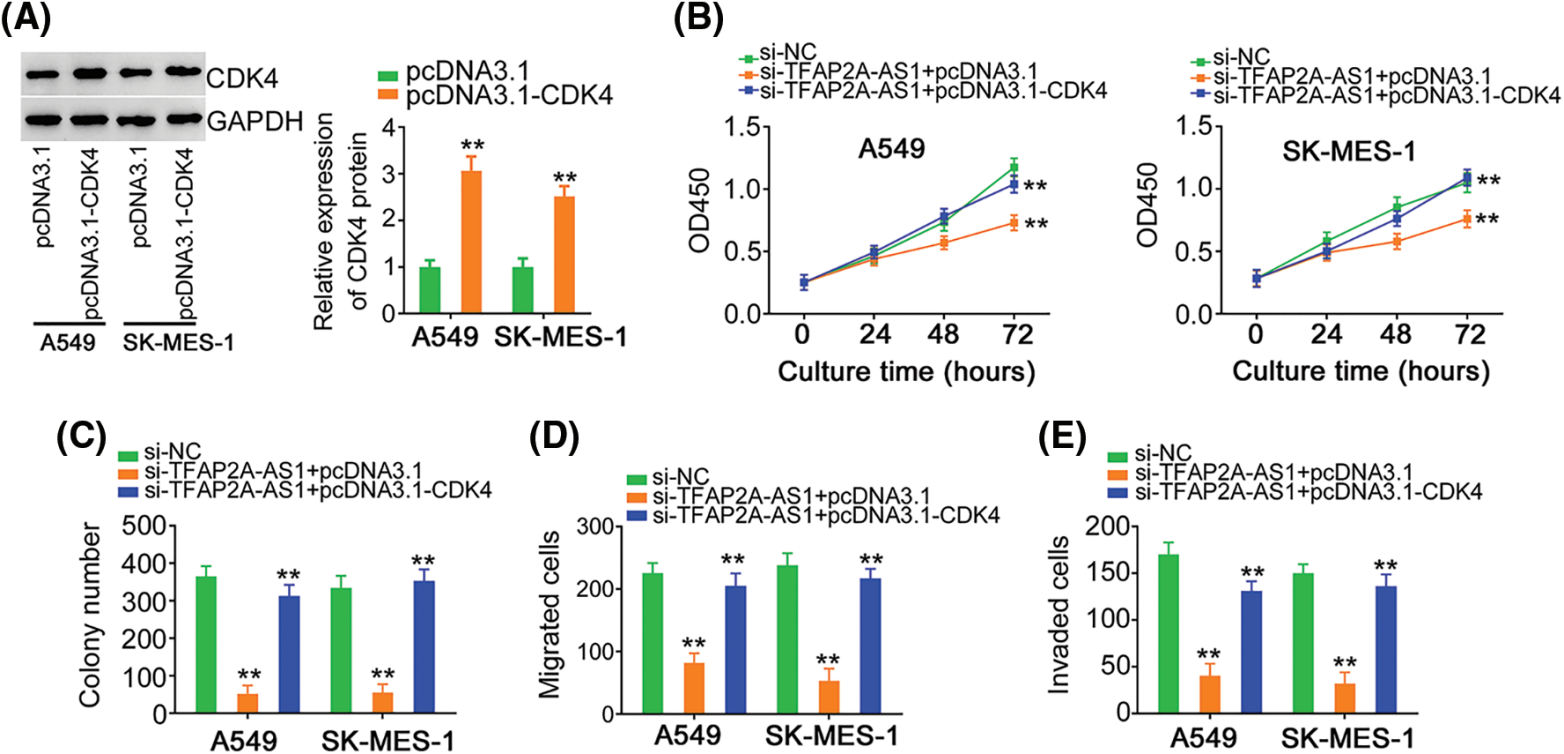
The effects of si-TFAP2A-AS1 on NSCLC cells are reversed by CDK4 upregulation. (A) CDK4 protein in pcDNA3.1-CDK4-transfected NSCLC cells was analyzed *via* western blotting. (B, C) NSCLC cells were received cotransfection of si-TFAP2A-AS1 and pcDNA3.1-CDK4 or pcDNA3.1. Detection of cell proliferation was implemented by CCK-8 and colony formation assays. (D, E) NSCLC cells treated as above described were subjected to Transwell assay for cell migration and invasion assessment. **p < 0.01 (n = 3).

Fig. 8A depicted the overexpression of CDK4 by pcDNA3.1-CDK4 in NSCLC cells. Cell proliferation and colony formation was hindered, which was retrieved by pcDNA3.1-CDK4 reintroduction (Figs. 8B and 8C). The ablation of TFAP2A-AS1 led to the diminution of cell migratory and invasive properties, whereas the effects were offset after treatment of pcDNA3.1-CDK4 (Figs. 8D and 8E). In a word, miR-584-3p/CDK4 axis is essential for the actions of TFAP2A-AS1 in NSCLC cells.

### TFAP2A-AS1 deficiency causes repression of tumor growth *in vivo*

**Figure 9:**
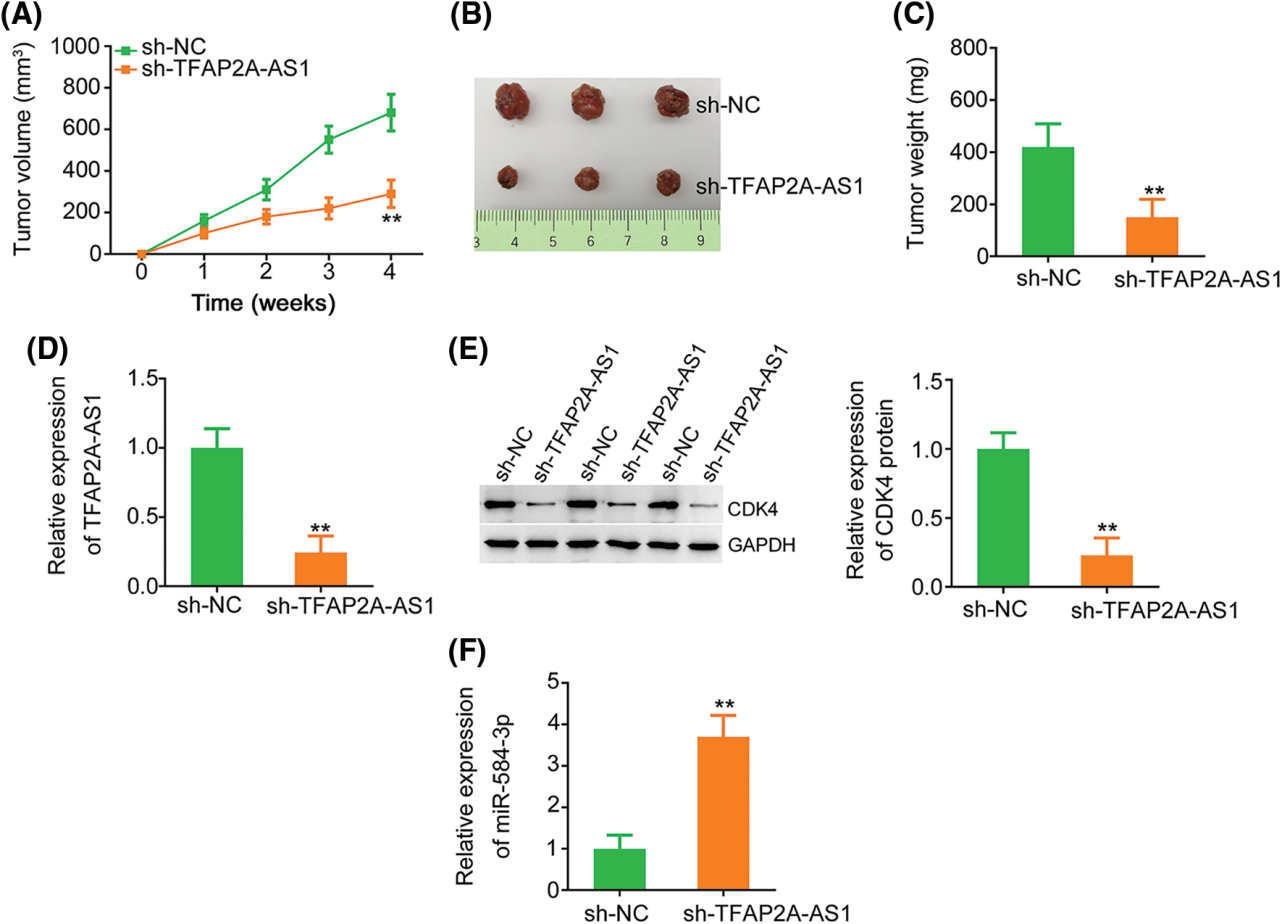
TFAP2A-AS1 depletion weakens tumor growth *in vivo*. (A) The volume of tumor xenografts were monitored till experiment ends, and growth curves were presented. (B) Representative images of excised tumor xenografts. (C) The weight of tumor xenografts was detected. (D) Total RNA was abstracted from tumor xenografts, and used for TFAP2A-AS1 level determination. (E) Western blotting was carried out to measure CDK4 protein level. (F) MiR-584-3p level was analyzed in tumor xenografts applying qRT-PCR. **p < 0.01 (n = 3).

We implemented tumor xenograft experiment to test the impact of TFAP2A-AS1 silencing on tumor growth *in vivo*. The volume (Figs. 9A and 9B) and weight (Fig. 9C) of tumor xenografts was reduced in the presence of sh-TFAP2A-AS1 stable transfection. Besides, TFAP2A-AS1 (Fig. 9D) and CDK4 (Fig. 9E) levels were declined, whereas miR-584-3p (Fig. 9F) level was upregulated in the TFAP2A-AS1-sienced tumors. That was to say, TFAP2A-AS1 ablation impedes tumor growth *in vivo*.

## Discussion

Ongoing progress of the high throughput gene sequencing technology implies the enrollment of lncRNAs in the realm of cancer genesis and progression [17,18]. LncRNAs have the ability to control the cancer-associated biological activities, thereby having aroused considerable interest over the past years [19]. Regarding tumor therapy, studying the contribution of lncRNAs to cancer pathogenesis is a promising research field, and can aid the identification of attractive therapeutic methods or antitumor drugs [20]. Therefore, we utilized diverse experiments to authenticate whether TFAP2A-AS1 can act as an essential modulator of NSCLC progression. What’s more, we explored the interaction among TFAP2A-AS1, miR-548-3p and CDK4.

Considerable evidences suggest that lncRNAs perform core functions in a broad spectrum of biological phenotype in NSCLC. For instance, lncRNAs CCAT1 [21], PART1 [22] and GACAT1 [23] are overexpressed in NSCLC, thus exerting pro-oncogenic actions. Instead, downregulated LINC00476 [24], MEG3 [25] and WT1-AS [26] in NSCLC are certified as anti-carcinogenic lncRNAs. TFAP2A-AS1 is downregulated in breast [27] and gastric [28] cancers, and performs tumor-inhibiting activities in controlling various aggressive phenotypes. But, the detailed roles of TFAP2A-AS1 in NSCLC still keep unclear by the public, so it is extremely urgent and must to make it definite. Herein, a notable overexpressed TFAP2A-AS1 was observed in both NSCLC tissues and cell lines. An increased TFAP2A-AS1 level displayed a negative correlation with the overall survival of patients with NSCLC. Loss-of-function approaches illustrated that the absence of TFAP2A-AS1 weakened NSCLC cell proliferation, colony formation, migration and invasion *in vitro*. Also, interference of TFAP2A-AS1 caused *in vivo* tumor growth suppression. Accordingly, these observations offer us with a novel perspective to comprehend NSCLC oncogenesis and progression, which may be helpful for treating this disease.

LncRNAs take part in the transcriptional and post-transcriptional processes and epidemic networks, establishing a complex regulation network [29]. The subcellular distribution of lncRNAs implicates the molecular events mediating their specific roles [30]. Regarding nucleic lncRNAs, they have the function of direct binding with different proteins. Unlike this, lncRNAs located in cell cytoplasm execute their regulatory actions through affecting the stability presented by mRNAs, which were happened via many methods. Among them, the ceRNA theory becomes the studying hot topic. LncRNAs possess miRNA response elements and can scavenge certain miRNAs from their target mRNAs, thereby generating lncRNA/miRNA/mRNA regulatory pathway.

The molecular events used by TFAP2A-AS1 in NSCLC were revealed in detail too. Initially, subcellular fractionation experiment was conducted, validating that both nucleus and cytoplasm saw TFAP2A-AS1 distribution, with the latter displaying a more part. Thus, we next searched the target of TFAP2A-AS1. MiR-584-3p shared complementary binding within TFAP2A-AS1. Next, luciferase reporter and RIP assay proved that TFAP2A-AS1 could negative regulated miR-584-3p as a ceRNA. The downstream effector of TFAP2A-AS1/miR-584-3p axis was also unveiled. Utilizing bioinformatic analysis and experimental confirmation, CDK4 was affirmed as the target of miR-584-3p, which was positively controlled by TFAP2A-AS1in a miR-584-3p-dependent manner. Altogether, we disclosed a novel ceRNA pathway in NSCLC, comprising TFAP2A-AS1, miR-584-3p and CDK4.

MiR-584-3p is lowly expressed in several human cancer types [31–33]. Yet, it is uncertain whether miR-584-3p is participated in the pathogenesis of NSCLC. In our current study, we also verified the downregulated miR-584-3p level in NSCLC, and the great contribution of miR-584-3p in the pathogenesis of NSCLC. Previously, miR-584-3p was confirmed to direct target Rho-associated, coiled-coil containing protein kinase 1 [31] in glioma, matrix metallopeptidase 14 [32] in gastric cancer, structure specific recognition protein 1 [33] in colorectal cancer. The target genes of miR-584-3p different in different tumors might be due to the tissue specificity of miRNAs in human cancers,

Located on 12q14.1, CDK4 is a silk/threonine protein kinase [34], and met the miR-584-3p target criteria to create its repressing activities in NSCLC. CDK4 performs carcinogenic actions during initation and progression during and is implicated in the regulation of tumor processes [14–16]. Herein, rescue function experiments were implemented, which corroborated that the anticancer activities of TFAP2A-AS1 deficient on the oncogenicity of NSCLC cells were reversed by downregulating miR-584-3p or overexpressing CDK4. Therefore, miR-584-3p/CDK4 axis is the downstream effector of TFAP2A-AS1 in NSCLC cells.

Our research had one limitation. The sample size of this study is small. We will collect more samples in our further experiments.

TFAP2A-AS is overexpressed in NSCLC and functions as a carcinogenic lncRNA to push cancer progression. Mechanistically, TFAP2A-AS1 exhibits its cancer-promoting role in NSCLC through adjusting the miR-584-3p/CDK4 axis. TFAP2A-AS1 positively controls CDK4 expression through sequestering miR-584-3p, forming a TFAP2A-AS1/miR-584-3p/CDK4 ceRNA pathway. The newly discovered pathway may offer inspirations for managing NSCLC.
